# Predictive relevance of optical coherence tomography indices in conjunction with visual acuity and surgical outcomes of idiopathic macular hole

**DOI:** 10.1016/j.heliyon.2024.e39261

**Published:** 2024-10-11

**Authors:** Sonu Kumar, G Nageswar Rao, Nidhi Sinha, Bhumika Rath, Sabya Sachi Pattanayak, Arttatrana Pal

**Affiliations:** a Department of Zoology, School of Life Sciences, Mahatma Gandhi Central University, Motihari, Bihar, 845401, India; b Department of Ophthalmology, Kalinga Institute of Medical Sciences, Kalinga Institute of Industrial Technology, Bhubaneswar, Odisha, 751024, India; c Vision Care, Center for Retina, Bhubaneswar, Odisha, 751024, India

**Keywords:** Optical coherence tomography indices, Idiopathic macular hole, Functional visual acuity, Anatomical success rate

## Abstract

Idiopathic macular hole (IMH) is a condition that arises from a combination of interactions among several forces on the fovea, remarkably from vitreous traction in the anteroposterior and tangential directions. Recent studies have highlighted the significance of microincision vitrectomy surgery, and IMH surgery was performed with minimal invasiveness, and visual improvement was an expected outcome. This study aimed to observe the pre-operative optical coherence tomography (OCT) indices correlated with visual acuity in the closure of IMH after surgery. Primarily, the findings were associated with clinical characteristics, including OCT indices, change in best corrected visual acuity (BCVA), clinical factors associated with IMH closure, and prognostic factors for the visual outcomes. This retrospective study included pre- and post-operative BCVA and OCT indices of 110 eyes with IMH. Each OCT variable was subjected to stepwise regression analysis regarding therapeutic factors that predict the need for IMH closure. Our results revealed that the hole form factor (HFF, r = 0.196), macular hole index (MHI, r = 0.669), and tractional hole index (THI, r = 0.085) had a positive correlation with visual acuity. However, basal hole diameter (BHD, r = −0.696) and minimum hole diameter (MHD, r = −0.407) showed a negative correlation. Out of them, HFF, MHI, BHD, and MHD were observed to be statistically significant (p < 0.05). The mean follow-up time was 149 ± 63.22 (85–300) days. The mean baseline BCVA was 0.75 ± 0.44 logMAR (Logarithm of the Minimum Angle of Resolution) units, which was improved to 0.29 ± 0.27 logMAR units at the final follow-up. The surgical success closure rate was 100 % among subjects with IMH. In conclusion, OCT indices were significant indicators of visual success rates in IMH, and OCT measurement could be employed as a single key index in predicting the IMH closure rate. Also, our findings suggested that OCT indices could be utilized as a safe and effective predictor of visual and anatomical outcomes in the case of IMH.

## 1 Introduction

Macular hole (MH) is a common cause of vision loss due to a full-thickness defect of retinal tissue in older persons, and the vast majority of cases are idiopathic [1]. Idiopathic macular holes (IMH) were thought to be caused by interactions between multiple pressures on the fovea, primarily from anteroposterior and tangential traction by the vitreous, and affect roughly 8.7 eyes per 100,000 every year [2,3]. Many studies have demonstrated that IMH affects people in their medium to late years of life and is not accompanied by other ocular diseases [[3], [4], [5]]. No permanent treatment against MH is available to date, and there are even advancements in ocular gene therapy delivery systems at subretinal and intravitreal regions, along with other advanced suprachoroidal techniques. The introduction of vitrectomy had shown that the surgical interventions were beneficial in 90 % of cases with full-thickness MH, promoting the anatomical closure of MH [[6], [7], [8], [9]]. Moreover, various modified surgical procedures are now being performed by ophthalmologists to restore the neurosensory retina to its normal anatomical position.

Recently, the focus has shifted from anatomical closure of the hole to achieving an excellent functional visual outcome. Optical coherence tomography (OCT) became an essential method for the pre-operative evaluation of MH that could create high-resolution retinal pictures. Specific MH characteristics might be pre-operative markers of future hole closure success. The minimum hole diameter (MHD) was reported to be the minimum linear dimension of the MH, and basal hole diameter (BHD) was reported as a linear dimension of the MH at the level of retinal pigment epithelium (RPE). The linear size of the MH was one of the most studied parameters for predicting post-operative visual outcomes. Studies have shown a negative correlation between the linear dimension of MH and visual outcomes. However, there was a difference in predictive performance regarding which dimension was more significant in terms of visual success [[10], [11], [12], [13]]. Further, the hole height (HH) was defined as the maximum distance between the RPE layer and the vitreoretinal interface, and a negative correlation was reported between HH and visual outcome [[14], [15], [16]]. The macular hole index (MHI) was defined as the ratio of HH to BHD and was reported to be positively correlated to post-operative visual acuity [17]. Earlier, a prospective study reported that a better visual outcome was observed in the subjects with MHI ≥0.5 than in those with MHI <0.5 [18]. However, few investigations reported no such correlation [19]. The tractional hole index (THI) was defined as the ratio of HH and MHD, and a retrospective study reported that THI >1.41 represents a predictive factor for good visual prognosis [17]. On the other hand, few studies have shown that the THI was not significant [16]. The OCT technique revolutionized the clinical practice of the ophthalmology field. However, the discrepancy between the studies regarding the significance of the pre-operative OCT indices in predicting final visual outcomes was a mystery yet to be resolved. In this retrospective study, we demonstrate the correlation between pre-operative OCT indices and postoperative visual outcomes in subjects undergoing IMH surgery with a modified inverted internal limiting membrane (ILM) flap technique.

## 2 Materials and methods

This retrospective investigation was conducted at the Vision Care, Center for Retina, Bhubaneswar, India. A retrospective chart review was performed for all subjects who underwent IMH surgery between February 18, 2018, and May 31, 2022, with a follow-up until October 31, 2022. The institutional review board of Kar Vision Eye Hospital, Bhubaneswar, India, approved this study (ECR/1630/Inst/OD/2021-A), which adhered to the tenets of the Declaration of Helsinki. Written informed consent was obtained from all subjects before any ophthalmic procedure. 110 subjects who fulfilled the criteria for inclusion and consented to participate in the investigation were included.

**Fig. 1:**
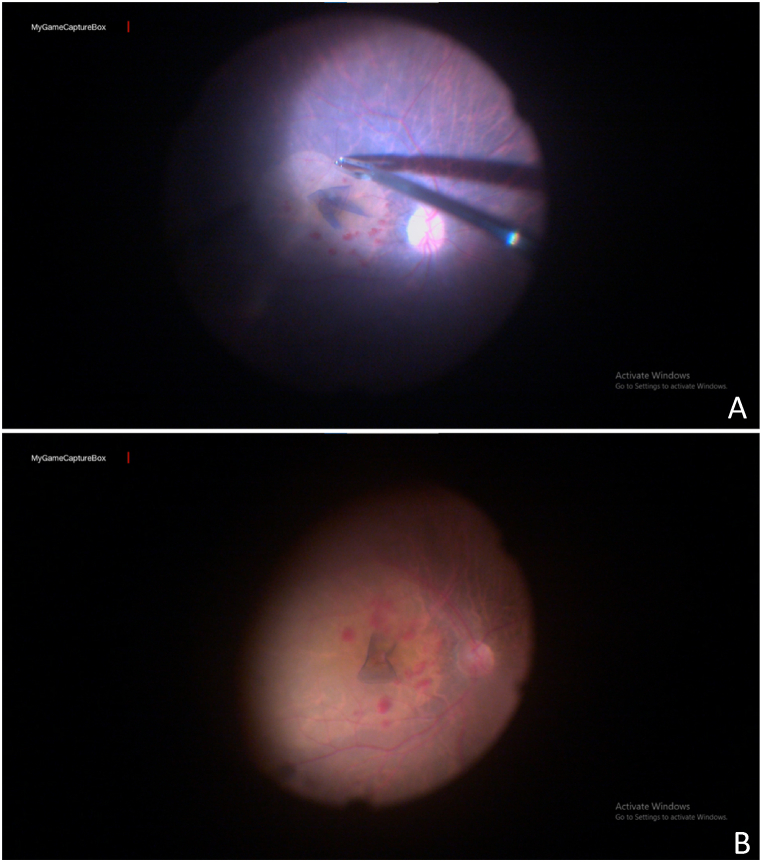
Images showing flower petals pattern inverted internal limiting membrane (ILM) flap (**A**). The desired residual ILM flaps after trimming extra length (**B)**.

After informed consent, all subjects underwent thorough ophthalmic examinations before IMH surgery. The surgery included subjects with full-thickness MH who underwent uncomplicated MH with no iatrogenic retinal break or vitreous hemorrhage. All ophthalmological interventions were performed by the same surgeon using a standard 3-port and 25-gauge pars plana vitrectomy based on a machine (Constellation® Vision System; Alcon Laboratories, Fort Worth, TX, USA). During IMH surgery, we considered very little trauma to the ocular surface, a shorter operation time, and reduced irrigation fluid. The ILM peeling was performed using an inverted ILM flap technique [20,21]. Our flower petal approaches were precisely based on the controlled formation of small flaps, preventing the rolling of a single, large flap over the MH and enabling the optic disc to serve as an anchor during traction on a petal flap that stabilized the retina. Briefly, the ILM peeling was completed using repeated pinch and peel techniques, creating tiny inverted flaps in a flower petal fashion (Fig. 1A). Excess flap lengths were cut using a vitrector with around 70 mmHg suction pressure so that the desired flaps automatically came together to fill the hole while doing fluid air exchange (Fig. 1B). Further, the epiretinal membranectomy was performed in subjects to remove the tangential traction when a pre-macular membrane was present. On the other hand, if no visible pre-macular membrane was observed in subjects, the ILM was peeled using brilliant blue-green (BBG) staining. After removing tangential traction around the MH, air-fluid exchange was performed, followed by sulfur hexafluoride (SF6) gas used as tamponade, and the face-down position was maintained for at least 2 days post-surgery. Moreover, a combined phaco vitrectomy was performed for subjects with cataracts and MH.

Multimodal imaging data such as fundus photography and OCT (Heidelberg Spectralis from Heidelberg Engineering Inc., Heidelberg, Germany) were recorded 24 h before surgery and 30 days and 90 days post-surgery. All pre-operative OCT scanning for this specific parameter of MH measurement was accomplished using the independent operators' caliber function of the OCT device. Postoperative and pre-operative corrected visual acuity (BCVA) corresponding to the scanned data were compared using OCT measurements. Each measurement was recorded three times for an average to minimize manual errors during the OCT screening, carried out by a single experienced operator. The Macular Cube 512 × 128 protocol was adopted to examine the entire macular geometry's horizontal and vertical orientations. Snellen visual acuity was used to record BCVA and further translated to the Logarithm of the Minimum Angle of Resolution (logMAR) scale for assessment. The BCVA was recorded before surgery and 30 days and 90 days post-surgery. Postoperative BCVA at the end of 90 days was considered to find the correlation between OCT index and visual acuity. Subjects with a history of trauma, other ocular pathology, and any intraocular surgery other than phacoemulsification of the lens were excluded from this study. Also, subjects with a follow-up duration of less than 90 days and whose pre-operative images were not clear due to media opacities were excluded. The patient's age, sex, and involved eyes were recorded. Various OCT indices like the size of the MH, BHD of the MH measured at the level of RPE, MHD, HFF (quotient of the sum of left and right arm lengths divided by BHD), MHI (ratio of HH to BHD), and THI (ratio of HH to MHD) were recorded.

Statistical analyses were performed using the IBM SPSS version 26.0 software (IBM Corp., Armonk, NY, USA). The Pearson correlation test assessed OCT index correlation with visual acuity. Repeated measures and analysis of variance changes in BCVA were analyzed. A linear regression analysis was carried out on each parameter. The P-value of ≤0.05 was considered statistically significant. The mean and standard deviation (SD) variables were indicated as mean ± SD.

## 3 Results

### 3.1 Sample characteristics

**Fig. 2:**
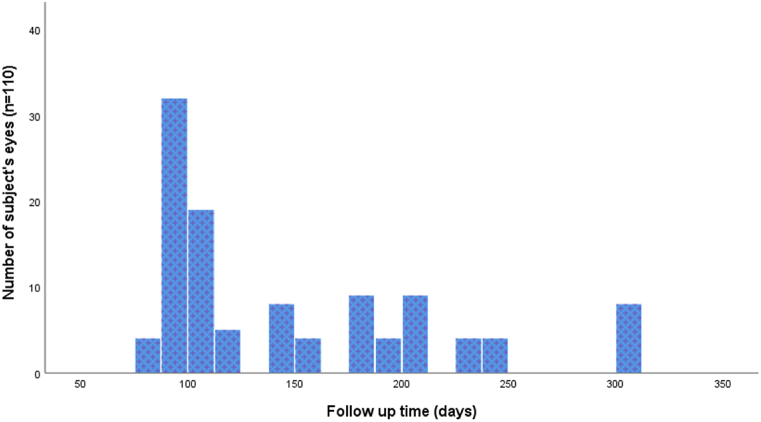
Graph showing the distribution of follow-up times among all macular hole (MH) subjects' eyes.

**Fig. 3:**
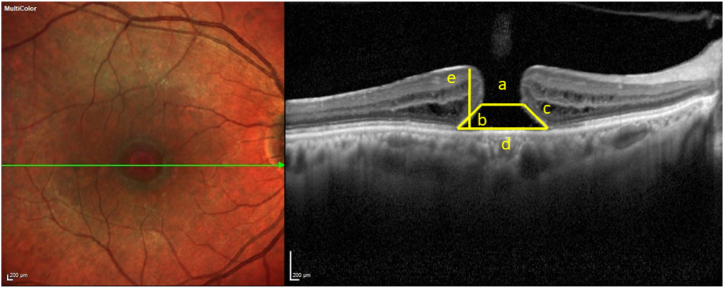
Dimensions of the macular hole (MH), as seen on optical coherence tomography (OCT), are depicted schematically**. a –** minimum hole diameter (MHD), **b –** left arm length, **c –** right arm length, **d –** basal hole diameter (BHD), **e –** hole height (HH), hole form factor (HFF)- (b + c)/d, macular hole index (MHI)**-** e/d, tractional hole index (THI) **–** e/a.

110 eyes from 110 subjects were included for IMH surgery with inverted ILM flap techniques. The mean age was 62.38 ± 5.70 years. 17 subjects ranged between 50 and 55 years, 23 subjects ranged between 55 and 60 years, 29 subjects ranged between 60 and 65 years, 36 subjects ranged between 65 and 70 years, and 5 subjects ranged between 70 and 75 years (Table 1). Of them, 60 (54.5 %) subjects were males and 50 (45.5 %) were females. The baseline follow-up duration was considered from the MH surgery date. The mean follow-up time was 149 ± 63.22 (85–300) days (Fig. 2). Our findings showed the demographic trends, pre-operative and post-operative BCVA, and various OCT indices like BHD of the MH measured at the level of RPE, MHD, HFF, MHI, and THI (Fig. 3). Further, the study showed that the mean post-operative visual acuity was 0.29 ± 0.27 logMAR. Of them, 22 subjects had a post-BCVA of 0.0 logMAR, 28 subjects had 0.1 logMAR, 19 subjects had 0.3 logMAR, 20 subjects had 0.4 logMAR, 17 subjects had 0.7 logMAR, and 4 subjects had 1.0 logMAR. Our findings showed that most subjects had poor visual acuity before surgery, and the maximum subjects had enhanced visual acuity after surgery. The descriptive statistical analysis of various variables is depicted in Table 1. The mean values of OCT indices of positive parameters HFF were (0.780 ± 0.18). Of them, 17 subjects had values 0.53–0.60, 17 subjects had values 0.61–0.70, 53 subjects had values 0.71–0.90, 19 subjects had values 0.91–1.10, and 4 subjects had values 1.31–1.36.

**Table 1:** The baseline characteristics and descriptive statistics of various variables such as hole form factor (HFF), macular hole index (MHI), tractional hole index (THI), basal hole diameter (BHD), minimum hole diameter (MHD) and hole height (HH) of optical coherence tomography (OCT) indices in subjects (N = 110 eyes) of IMH surgery.

Parameters (N = 110)	Mean	SD	Range	Minimum	Maximum
**HFF**	0.78	0.18	0.83	0.53	1.36
**MHI**	0.45	0.12	0.52	0.21	0.73
**THI**	1.17	0.65	2.99	0.49	3.48
**BHD (*μ*m)**	972.33	264.03	1044.00	330.00	1374.00
**MHD (*μ*m)**	434.22	166.99	722.00	129.00	851.00
**HH (*μ*m)**	432.74	102.70	531.00	184.00	715.00
**AGE (Years)**	62.38	5.708	21.00	50.00	71.00

The baseline subjects (N = 110 eyes) characteristics and descriptive statistics of various variables, including HFF, MHI, THI, BHD, MHD, and HH of OCT indices, were depicted in Table 1, Table 2. The mean value of the OCT index MHI was 0.454 ± 0.12. Out of them, 12 subjects had values ranged 0.21–0.30, 28 subjects had values ranged 0.31–0.40, 28 subjects had values ranged 0.41–0.50, 33 subjects had values ranged 0.51–0.60, and 9 subjects had values ranged 0.61–0.73. The mean value of the OCT index THI was 1.17 ± 0.65. Of them, 20 subjects had values 0.49–0.70, 37 subjects had values 0.70–1.00, 21 subjects had values 1.00–1.20, 22 subjects had values 1.30–1.90, and 10 subjects had values 1.90–3.48. The average mean value of the negative correlation OCT parameter BHD was 972.33 ± 264.03 μm. Out of them, 8 subjects had values of 330–500 μm, 14 subjects had values of 600–800 μm, 42 subjects had values of 800–1000 μm, 13 subjects had values of 1000–1200 μm, and 33 subjects had values of 1200–1374 μm. The average mean value of OCT parameter MHD was 434.22 ± 166.99 μm. Out of them, 8 subjects had values 129–200 μm. 24 subjects had values from 200 to 350 μm, 21 subjects had values 350–450 μm, 49 subjects had values 450–650 μm, and 8 subjects had values 700–851 μm. The average mean value of OCT parameter HH was 432.74 ± 102.70 μm. Out of them, 8 subjects had values 184–250 μm, 8 subjects had values 300–350 μm, 44 subjects had values 350–450 μm, 45 subjects had values 450–550 μm, and 5 subjects had values 700–715 μm.

**Table 2:** The baseline pre-operative clinical characteristics of various optical coherence tomography (OCT) metrics such as hole form factor (HFF), macular hole index (MHI), tractional hole index (THI), basal hole diameter (BHD), minimum hole diameter (MHD) and hole height (HH) of optical coherence tomography (OCT) indices in subjects (N = 110 eyes) of IMH surgery.

HFF	N (110)	MHI	N (110)	THI	N (110)	BHD (***μ***m)	N (110)	MHD (***μ***m)	N (110)	HH (***μ***m)	N (110)	AGE (Years)	N (110)
0.53–0.60	17 (15.45 %)	0.21–0.30	12 (10.9 %)	0.49–0.70	20 (18.18 %)	330–500	8 (7.27 %)	129–200	8 (7.27 %)	184–250	8 (7.27 %)	50–55	17 (15.45 %)
0.61–0.70	17 (15.45 %)	0.31–0.40	28 (25.45 %)	0.70–1.00	37 (33.63 %)	600–800	14 (12.72 %)	200–350	24 (21.81 %)	300–350	8 (7.27 %)	55–60	23 (20.09 %)
0.71–0.90	53 (48.18 %)	0.41–0.50	28 (25.45 %)	1.00–1.20	21 (19.09 %)	800–1000	42 (38.18 %)	350–450	21 (19.09 %)	350–450	44 (40 %)	60–65	29 (26.36 %)
0.91–1.10	19 (17.27 %)	0.51–0.60	33 (30.0 %)	1.30–1.90	22 (20.0 %)	1000–1200	13 (11.81 %)	450–650	49 (44.54 %)	450–550	45 (40.9 %)	65–70	36 (32.72 %)
1.31–1.36	4 (3.63 %)	0.61–0.73	9 (8.18 %)	1.90–3.48	10 (9.09 %)	1200–1374	33 (30.0 %)	700–851	8 (7.27 %)	700–715	5 (4.54 %)	70–71	5 (4.54 %)

### 3.2 Correlation between specific OCT indices and postoperative visual acuity

**Fig. 4:**
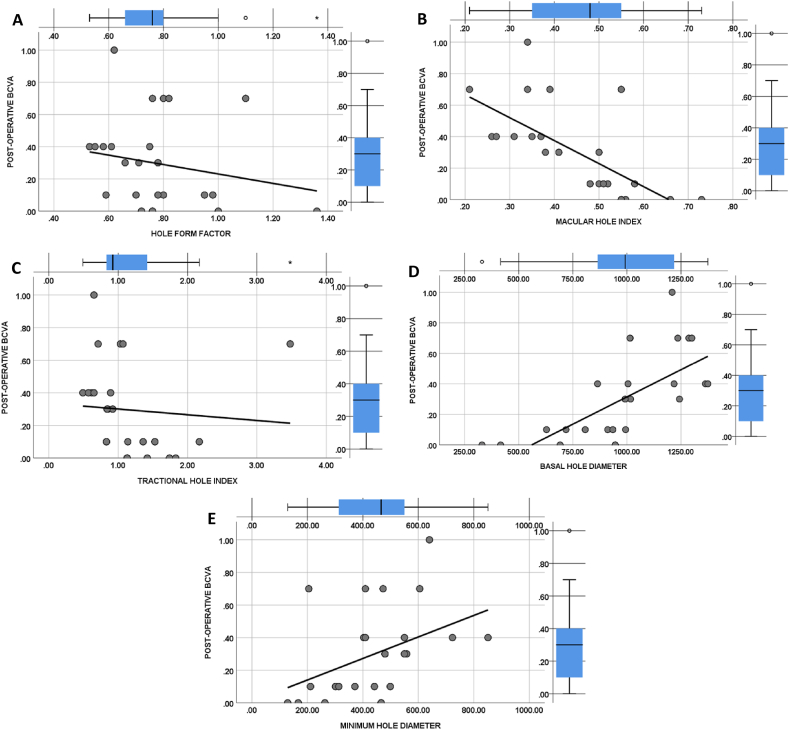
Graph demonstrating the co-relationship between various optical coherence tomography (OCT) indices metrics and postoperative visual acuity; Best corrected visual acuity (BCVA) in logMAR units; Basal hole diameter (BHD) in μm units; Minimum hole diameter (MHD) in μm units.

Our primary findings highlighted the demographic characteristics that could be applied to predict the link between visual outcomes and baseline OCT parameters to close MH. Further, Pearson correlation coefficient regression analysis showed a positive correlation between HFF (r = 0.196, p = 0.041) (Fig. 4A), MHI (r = 0.669, p = 0.000) (Fig. 4B), THI (r = 0.085, p = 0.37) (Fig. 4C), and visual acuity. Meanwhile, BHD (r = −0.696, p = 0.000) (Fig. 4D) and MHD (r = −0.407, p = 0.000) (Fig. 4E) showed a negative correlation. The HFF (r = 0.196, p = 0.041), MHI (r = 0.669, p = 0.000), BHD (r = −0.696, p = 0.000), and MHD (r = −0.407, p = 0.000) were observed to be statistically significant (P < 0.05). Of them, it was noticed that MHI had a strong positive correlation and HFF had a weak positive correlation with post-operative visual acuity. Further, it was noticed that OCT parameter MHD had a moderately negative correlation (r = −0.407) and BHD had a strong negative correlation (r = −0.696) with post-operative visual acuity. Moreover, our study found no significant relationship between age and postoperative visual acuity (P = 0.4).

### 3.3 Visual and anatomical outcomes

**Fig. 5:**
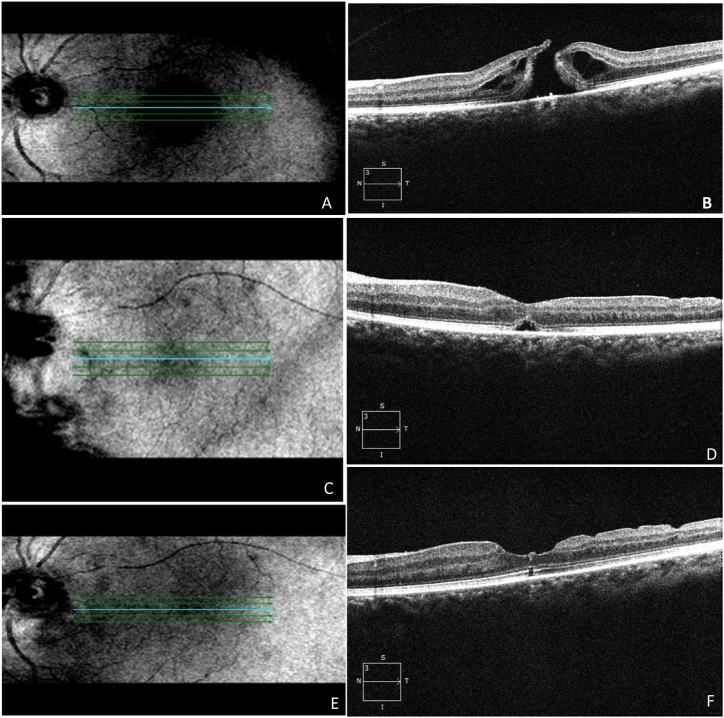
A representative image of a vitrectomy with an internal limiting membrane (ILM) peeling for an idiopathic macular hole (IMH). Hole form factor (HFF = 0.98), macular hole index (MHI = 0.52), tractional hole index (THI = 1.53), basal hole diameter (BHD = 911 μm), and minimum hole diameter (MHD = 313 μm) were noted using optical coherence tomography (OCT) images showing macular hole (**A**,**B).** Best corrected visual acuity (BCVA) was 0.6 logMAR before the vitrectomy. Subsequently, 18 days after the vitrectomy, the OCT scan showed improvement in hole closure rate, and the presence of a flap was observed (**C,D**). The idiopathic macular hole (IMH) remained closed after 123 days post-vitrectomy, and her BCVA was 0.1logMAR (**E,F).**

**Fig. 6:**
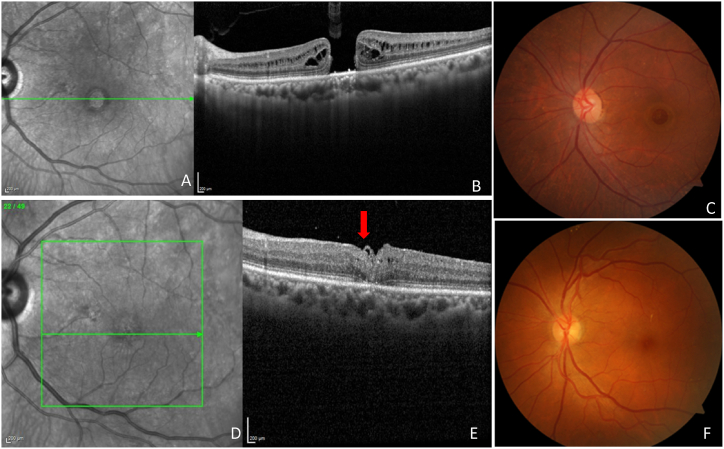
A representative image of a vitrectomy with an internal limiting membrane (ILM) peeling for an idiopathic macular hole (IMH). Hole form factor (HFF = 0.53), macular hole index (MHI = 0.31), tractional hole index (THI = 0.49), basal hole diameter (BHD = 1363 μm), and minimum hole diameter (MHD = 851 μm), were noted using optical coherence tomography (OCT) showing fundus images of idiopathic macular hole (IMH) (**A,B,C**). The best corrected visual acuity (BCVA) was 0.4 logMAR before the vitrectomy. Subsequently, 120 days after vitrectomy, the OCT scan improved the hole closure rate. The IMH remained closed after 120 days post-vitrectomy, and their BCVA was the same at 0.4 logMAR (**D,E,F**), and the flap was marked by a red arrow (E).

**Fig. 7:**
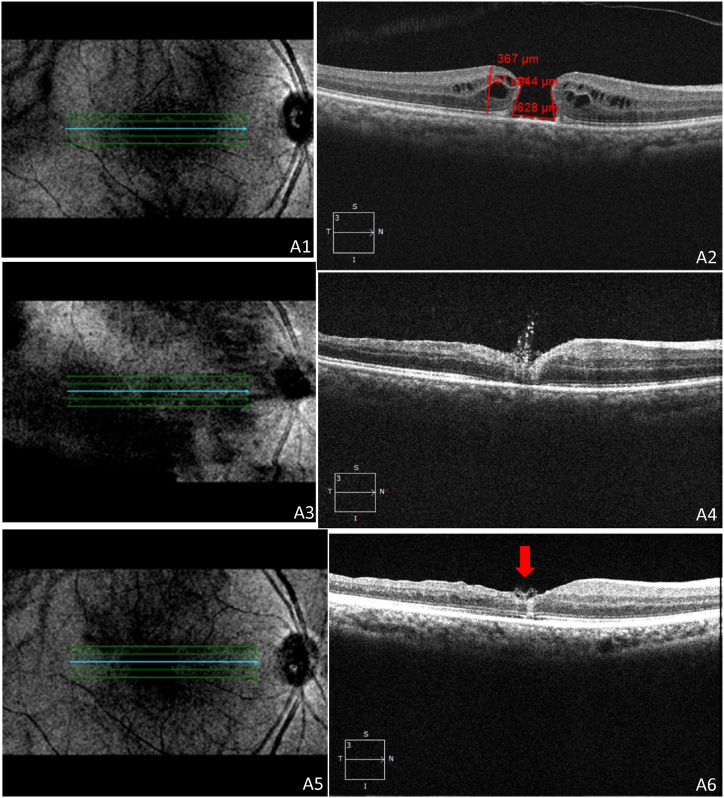
A representative image of a vitrectomy with an internal limiting membrane (ILM) peeling for an idiopathic macular hole (IMH). Hole form factor (HFF = 0.78), macular hole index (MHI = 0.58), tractional hole index (THI = 0.67), basal hole diameter (BHD = 628 μm), and minimum hole diameter (MHD = 367 μm) were noted before surgery by using optical coherence tomography (OCT) (**A1,A2**). Best corrected visual acuity (BCVA) was 0.6 logMAR before the vitrectomy. After 27 days of vitrectomy surgery, the OCT scan showed improvement in the hole closure rate (**A3,A4**). The IMH remained closed after 300 days post-vitrectomy, and BCVA was 0.1 logMAR (**A5,A6**) and the flap was marked by a red arrow (A6).

**Fig. 8:**
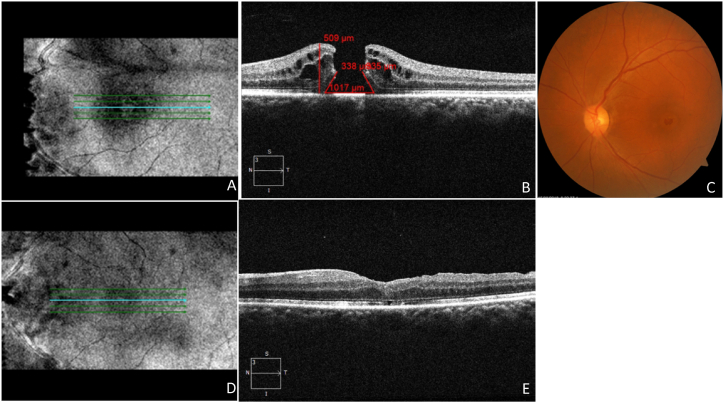
A representative image of a vitrectomy with the internal limiting membrane (ILM**)** peeling for an idiopathic macular hole (IMH). Hole form factor (HFF = 0.66), macular hole index (MHI = 0.5), tractional hole index (THI = 0.92), basal hole diameter (BHD = 1017 μm), minimum hole diameter (MHD = 550 μm), was noted using optical coherence tomography (OCT) images showing macular hole (MH) (**A,B**). Fundus images before surgery were recorded (**C).** Best corrected visual acuity (BCVA) was 0.7 logMAR before the vitrectomy. Subsequently, 36 days after the vitrectomy the OCT scan showed improvement in the hole closure rate. The IMH remained closed after 180 days post-vitrectomy, and BCVA was 0.3 logMAR (**D,E**).

The secondary outcomes included the changes in BCVA. The correlation between pre- and post-operative BCVA is depicted in Table 3. Our study also illustrated the prognostic factors, which confirmed that subjects with MHI ≥0.5, HFF ≥0.9, and THI ≥1.41 obtained better visual acuity of ≤0.3 logMAR, and subjects with MHI <0.5, HFF <0.9, and THI <1.41 obtained poor visual acuity of >0.3logMAR. The subjects with MHI ≥0.5, HFF ≥0.9, and THI ≥1.41 with better change in visual acuity from 0.6 to 0.1 logMAR were shown in Fig. 5A-F. The subjects with MHI <0.5, HFF <0.9, and THI <1.41 with poor change in visual acuity were shown in Fig. 6A-F. Moreover, our study revealed that 69 subjects (62.72 %) retained ≤0.3logMAR value of visual acuity, whereas 41 (37.28 %) subjects retained >0.3 logMAR value of visual acuity post-operatively. Only 4 subjects (3.6 %) retained a 1.0 logMAR visual acuity value postoperatively. The better visual outcomes from 0.6 to 0.1 logMAR were recorded with relatively lower values of BHD and MHD. The vitrectomy with an inverted ILM flap technique for an IMH, HFF = 0.78, MHI = 0.58, THI = 0.67, BHD = 628 μm, and MHD = 367 μm were measured before surgery by using OCT (Fig. 7A1, A2). The BCVA was 0.6 logMAR before vitrectomy. After 27 days of vitrectomy, the OCT scan improved the hole closure rate (Fig. 7A3, A4). The IMH remained closed after 300 days post-vitrectomy, and BCVA was 0.1 logMAR (Fig. 7A5, A6). The visual outcomes from 0.7 to 0.3 logMAR showed relatively higher values of BHD and MHD. The vitrectomy with inverted ILM flap technique for an IMH, HFF = 0.66, MHI = 0.5, THI = 0.92, BHD = 1017 μm, MHD = 550 μm, was measured using OCT (Fig. 8A and B). As shown in Fig. 8C, a fundus image was recorded before surgery, and the BCVA was 0.7 logMAR. After 36 days of vitrectomy, the OCT scan improved the hole closure rate. The IMH remained closed after 180 days post-vitrectomy, and BCVA was 0.3 logMAR (Fig. 8D and E). The mean baseline BCVA was 0.75 ± 0.44 logMAR units, while the mean post-operative BCVA at the final follow-up was 0.29 ± 0.27 logMAR units.

**Table 3:** Comparison between pre-operative and post-operative best corrected visual acuity (BCVA) in subjects of IMH surgery. CF = Counting fingers; logMAR = logarithm of the minimum angle of resolution.

		POST-OPERATIVE BCVA (logMAR)						Total (N)
		0.0	0.1	0.3	0.4	0.7	1.0	
**PRE-OPERATIVE BCVA (logMAR)**	**0.4**	4	0	0	4	0	4	12
	**0.6**	14	24	15	12	5	0	70
	**0.7**	0	4	0	0	8	0	12
	**1.0**	0	0	0	4	0	0	4
	**CF**	4	0	4	0	4	0	12
**Total (N)**		22	28	19	20	17	4	110

**Fig. 9:**
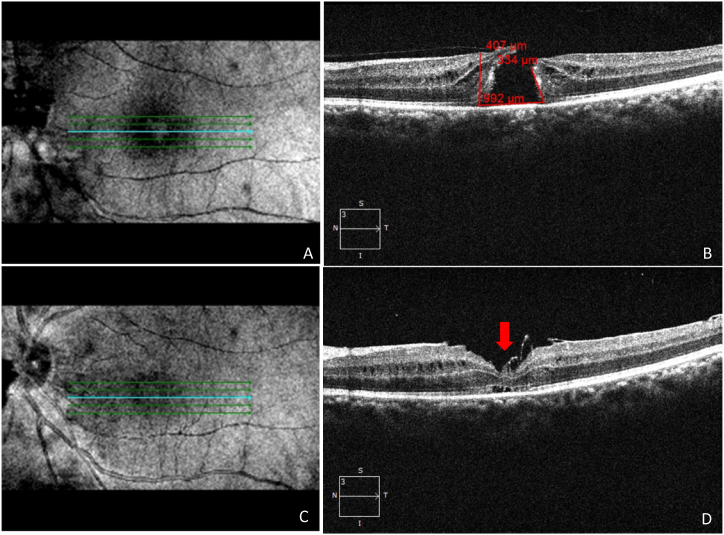
A representative image of a vitrectomy with the internal limiting membrane (ILM) peeling for an idiopathic macular hole (IMH). Hole form factor (HFF = 0.78), macular hole index (MHI = 0.41), tractional hole index (THI = 0.84), basal hole diameter (BHD = 992 μm), minimum hole diameter (MHD = 480 μm) were noted using optical coherence tomography (OCT) images showing IMH **(A,B**). Best corrected visual acuity (BCVA) was 0.6 logMAR before the vitrectomy. Subsequently, 12 days after the vitrectomy, the OCT scan showed improvement in hole closure rate, and a flap was observed. The IMH remained closed post-vitrectomy, and BCVA was 0.3 logMAR (**C,D)** and the flap was marked by a red arrow (**D**).

The V-shaped and U-shaped closure was our criterion for effective anatomic closure. All 110 eyes retrieved a complete hole closure, and none were in an open state, according to an interpretation of the multimodal imaging data (Table 4). Further, our findings supported the idea of the clinical factors linked with MH closure and indicated that the subjects with MHI ≥0.5, HFF ≥0.9, and THI ≥1.41 had a higher success rate of hole closure with better visual outcomes in a single surgical approach. However, subjects with MHI <0.5, HFF <0.9, and THI <1.41 had a lower success rate of hole closure with poor visual outcomes (Table 4). Further, to evaluate the efficiency of surgery based on BCVA, we characterized mainly three statuses of BCVA. First, the subjects' pro-BCVA increased by more than two lines on the visual acuity chart. These findings confirmed an improvement in BCVA. Second, they fluctuated by one line, indicating no change in BCVA. Third, they decreased by more than two lines, indicating a worsening of BCVA. Further, functional efficacy was assessed when all subjects with anatomical closure were confirmed, and there was a two-line improvement in Snellen's visual acuity. Further, we observed that hole-closed eyes obtained a better enhancement in eyesight. Along with this, we also observed clinical features on OCT images that revealed 13/110 (11.81 %) of subjects with flap and 9/110 (8.18 %) with vitreomacular traction (VMT). The change in visual acuity in subjects with a flap was shown in Fig. 9A-D. We also found a marginally higher closure rate and better visual acuity in subjects with VMT than in subjects without VMT.

**Table 4:** The Pearson correlation analysis between optical coherence tomography (OCT) indices and prognostic factors including best corrected visual acuity (BCVA), hole form factor (HFF), macular hole index (MHI), tractional hole index (THI), basal hole diameter (BHD), minimum hole diameter (MHD), logarithm of the minimum angle of resolution (logMAR) in subjects of IMH surgery. Level of significance, ∗p < 0.05.

OCT Indices	Pearson coefficient	p-value	Correlation with visual acuity	VALUE FOR GOOD PROGNOSIS	BCVA GAIN (logMAR unit)	IMH STATUS
	r	p				
**HFF**	0.196	0.041∗	Positive	≥0.9	0.0 (n = 22)	CLOSED
**MHI**	0.669	0.000**∗**	Positive	≥0.5	0.1 (n = 28)	CLOSED
**THI**	0.085	0.37	Positive	≥1.41	0.3 (n = 19)	CLOSED
**BHD**	−0.696	0.000∗	Negative	–	–	–
**MHD**	−0.407	0.000**∗**	Negative	–	–	–

## 4 Discussion

The dimensions and geometry of the MH are fundamental in deciding the outcome of MH surgery [22]. Even though advancements in macular surgery procedures have been shown to enhance visual acuity in most instances, MHs could provide a difficult surgical challenge and the danger of many recurrences. Furthermore, despite the surgical success, some still have metamorphopsia or small scotomas that cannot be corrected, generating dissatisfaction for patients and surgeons [8,23]. As a result, clinical assessments are crucial in understanding surgical success rates. Our study investigated visual recovery after IMH surgery and the related prognostic factors. Many studies have reported that the closure success rate after IMH surgery was less than 100 % [7,24]. In this study, we achieved a closure success rate of 100 % using a standard 25-gauge vitrectomy assisted with inverted ILM flap techniques. Earlier studies have reported that 5 %–9.5 % of eyes suffered late reopening of initially closed MH [[25], [26], [27]]. In contrast, our findings revealed that no eyes experienced a late MH reopening following 3 months of successful closure. However, the key findings are precisely incorporated and supported by our flower petal approaches, which facilitate a controlled formation of small flaps, prevent the rolling of both a single and large flap over the MH, and enable the optic disc to serve as an anchor during traction on the petal flaps that stabilized the retina.

Earlier studies have shown in MH surgery that 39 eyes were addressed with SF6 and 39 eyes were addressed with perfluoro ethane (C2F6) that accomplished anatomical closure rates of 87 % and 90 %, respectively [28]. Also, the mean visual acuity improved to 0.38 logMAR from 0.78 logMAR in the SF6 group and to 0.44 logMAR from 0.81 logMAR in the C2F6 group at 6 months following the surgery [28]. However, we observed significant improvement in the mean value to 0.29 logMAR from 0.75 logMAR within the 3 months with SF6-assisted surgery, along with a 100 % closure rate in 110 eyes. Studies have reported that in a 23-gauge vitrectomy, vision acuity stabilized by 6 months following MH surgery [29]. Another study has shown continuous visual improvement after MH surgery following subjects up to 96 months [30]. However, our findings revealed that the vision acuity stabilized by 9 months in a 25-gauge vitrectomy assisted with modified inverted ILM flap techniques. More importantly, our study reported that during IMH surgery, significantly less trauma to the ocular surface, a shorter operation time, and a reduced amount of irrigation fluid were considered to be reasons for the shorter time to visual recovery in the 25-gauge vitrectomy subjects.

The emergence of OCT has allowed for a more realistic image of MH architecture, measurement, and study of the process's pathophysiology. On the other hand, OCT was frequently utilized to measure and monitor MH features such as MHI, HFF, and THI. Also, OCT was utilized in easing therapy, decision-making, and the expected surgical outcome [31]. Many studies have reported the link between BCVA and pre-operative morphology assessed by OCT in eyes with MH, such as the basal and minimum MH size, external limiting membrane (ELM) height, and maximum retinal thickness [32]. Compared to MHI and HFF, the area ratio factor (ARF) adequately represented the three-dimensional properties of MH, achieving higher sensitivity and specificity. As a result, ARF may be the most influential parameter for predicting visual prognosis in MH surgery [33]. The imaging of foveal microstructure has significantly enhanced with newer high-resolution OCT devices. Recent studies have shown that the postoperative state of the inner segment-outer segment junction correlates strongly with the visual results after MH surgery and any disturbance in this layer may be linked with inferior visual outcomes [14].

Similarly, OCT indices BHD, MHD, THI, and MHI reported a significant correlation with BCVA at 3 months following MH surgery [17]. The effectiveness of the MH angle in predicting the success of MH closure was examined in another pre-operative study. However, no correlation between MH angle and closure was statistically significant [34]. A few studies observed that MHI did not significantly predict anatomical success. However, our findings strongly confirmed that MHI is a significant anatomical success predictor. In another study, OCT metrics MHI (P ≤ 0.05) were taken as a significant indicator for better MH surgical outcomes [29]. These results were also comparable to those of our study. However, the sample size was smaller in the previous study, and the surgical techniques employed indocyanine green (ICG) dye, which was quite different from our studies, as the methodology involved a more significant number of samples and brilliant blue G (BBG) dye without the consideration of specific stages of IMH. Thus, our study strongly suggests that MHI predictive value could be applied in different stages of IMH, as assisted by our modified inverted ILM flap techniques.

An earlier study reported a small sample size, including 46 subjects, and the use of ICG dye during IMH surgery. The OCT index MHI and THI were considered reliable indicators in predicting better outcomes of MH surgery [17]. These results demonstrated that, along with MHI, THI was also significantly correlated with visual acuity at post-operative 3 months. Our study also suggested that MHI has a strong positive and significant correlation with post-operative visual acuity, whereas THI has no significant correlation with visual acuity. Moreover, studies have shown that, in 94 subjects, better post-operative visual outcome was correlated with a higher value of HFF [35]. Similar to these findings, our results confirmed that post-operative visual outcomes were significantly related to HFF with a weak correlation compared to MHI. The cause of the discrepancy between these investigations could be using various software and OCT machinery models and surgical procedures. Previously, it was shown that pre-operative measurement of BHD and MHD with OCT could be reported as a predictive factor for postoperative visual prognosis. Similarly, several studies have presented a correlation between postoperative visual acuity and OCT measurements of the apical rather than BHD of the MH [11,35]. However, our findings showed a significantly negative correlation between postoperative visual acuity and OCT metrics, such as BHD and MHD. Previously, a retrospective analysis of 50 eyes reported a 10 % deterioration in visual acuity for every 26 μm increment in the BHD [17]. However, no such pattern was observed despite incorporating BHD extremes extending from 300 to 1400 μm in our analysis.

Many studies have demonstrated that the MH area index measurement could be important in predicting the hole closure in IMH [36]. Although it is difficult, attempts could be made to explain the discrepancy between the anatomic and functional results by examining the abnormalities found in the macular morphology as depicted by OCT. However, some visual outcomes still could not be predicted by morphological characteristics [32,37]. Previously, studies have reported that the defined technique could attain acceptable anatomical closure. Several clinical studies have recently suggested that the inverted ILM flap techniques achieved better anatomical and visual outcomes than standard ILM peeling [38]. Several OCT parameters have been linked to effective MH closure and improved visual acuity, including MH width, height, volume, ellipsoid zone (EZ), changes, and ELM characteristics. However, data were scarce on the entire evaluation of various OCT characteristics following successful surgical repair, and it was unclear if a single parameter at a time point on OCT corresponds best to visual results [39]. However, our study considered more than one variable at one time, including HFF, MHI, THI, BHD, and MHD, with significant prognostic value to predict visual and anatomical success in a large sample size. Earlier reports indicated that several biomarkers determined from OCT were effective indicators of progression, severity, and visual outcome. Further, ICG dye was formerly used to stain ILM for peeling. However, some researchers have found retinotoxicity [40]. Our investigation showed excellent staining with BBG dye equivalent to ICG without any subjects with retinotoxicity. Additionally, we applied BBG dye under a balanced salt solution, significantly minimizing the surgical procedure time compared to ICG.

A case series revealed no significant relationship between MH size and the presence or absence of VMT. Also, their findings suggested that the presence of VMT did not affect hole closure rates and hole size [41]. In our study, a similar pattern of findings was seen in 9/110 (8.18 %) subjects, and it was noticed that even in the presence of VMT, the subjects retained hole closure. In addition, it was also observed that a flap did not affect the hole closure rate in a few subjects during the follow-up duration. Moreover, our study confirmed the feasibility of OCT imaging during MH surgery. Our study findings also provided significant evidence and reported that the hole closure in the early postoperative period might be highly predicted by utilizing multiple OCT variables. In this direction, a further controlled comparison analysis is required to check these findings and establish their efficacy compared to the standard treatment.

Several studies have shown that OCT data from MH eyes were used to predict visual outcomes such as MH size, MHI, BHD, and other OCT variables [19]. Among these, it was claimed that the MHI was a significant predictor of visual prognosis after MH surgery [42]. However, despite the single factor alone, our study observed that other OCT indices might be significant indicators of postoperative visual acuity. It revealed a positive correlation between postoperative visual acuity and HFF, MHI, and THI and a negative correlation with BHD and MHD. The study showed that visual acuity gains take a long time in elderly people [43]. Conversely, neither age nor gender was significantly correlated with postoperative BCVA in our investigations (P = 0.6). The OCT parameters that could mirror the prognosis of the operation and its visual result have grown in popularity among MH publications, and they were widely considered a reliable baseline predictor for the medical outcome, specifically in MH subjects. The identification of the OCT indices obtained in the present study was simple to apply, and, thereby, the better outcome of the surgical procedure could be determined pre-operatively. However, our study has some limitations due to its retrospective nature and inconsistent subject's variable mean follow-up time of 149 ± 63.22 (range: 85–300) days. This study population was confined to Eastern India only. The same surgeon performed surgery on all subjects, so the results may be limited. Also, the irregular subject's follow-up time and inadequacy of immediate post-surgery OCT may have led to delays in early diagnosis of MH. Future research should consider these aspects while adapting these conclusions to clinical settings. A larger sample size, multicenter investigation, and meta-analysis might be required to determine whether all these pre-operative OCT indices of MH act as effective and significant predictors for postoperative BCVA.

## 5 Conclusion

The findings of this study showed better visual outcomes following IMH surgery and the related prognostic factors, with an anatomical success rate using vitrectomy and a modified inverted ILM flap technique. In IMH surgery, OCT indices are significant indicators of visual success rates, and OCT measurement might be employed as a single key index in predicting hole closure. The measurable values of HFF, MHI, BHD, and MHD were observed as significant correlations with visual acuity and predictive factors for better visual prognosis following IMH surgery. Our findings suggested that OCT indices could be adopted as a safe and efficient predictor of visual and anatomical outcomes in the case of IMH. Moreover, OCT indices are good pre-operative predictors of postoperative functional and visual outcomes following surgery. A larger sample size is required to determine the baseline markers could be applied to numerous diagnostic criteria of IMH. Further, these predictive parameters for hole closure based on OCT variables offer a great opportunity for image-based decision-making in postoperative care, with future confirmation as well as unique insights into the role that OCT measurements might play a major role in visual and anatomical outcomes.

## CRediT authorship contribution statement

**Sonu Kumar:** Writing – original draft, Formal analysis, Data curation. **G Nageswar Rao:** Writing – original draft, Methodology, Formal analysis, Data curation. **Nidhi Sinha:** Methodology, Formal analysis. **Bhumika Rath:** Methodology, Formal analysis. **Sabya Sachi Pattanayak:** Methodology, Formal analysis. **Arttatrana Pal:** Writing – review & editing, Writing – original draft, Methodology, Formal analysis, Data curation, Conceptualization.

## Data availability statement

Data will be made available from the corresponding author on request.

## Funding

This research received no specific grant from any funding agency in the public, commercial or not-for-profit sectors.

## Declaration of competing interest

All authors have declared no conflict of interest and substantially contributed for this work.
